# High unchanged incidence of diabetic ketoacidosis between 2000 and 2009 in Auckland children

**DOI:** 10.1186/1687-9856-2013-S1-O32

**Published:** 2013-10-03

**Authors:** SW Cutfield, J Derraik, C Jefferies, PL Hofman, WS Cutfield

**Affiliations:** 1 Liggins Institute, University of Auckland, New Zealand; 2 Starship Childrens Hospital, Auckland, New Zealand

## Background

Diabetic ketoacidosis (DKA) is a potentially life threatening complication of initial presentation with type 1 diabetes mellitus (T1DM). Annual DKA incidence data has been reported in selected populations in many studies, however few studies have reported the trend in DKA incidence over time in an unselected representative childhood population.

## Aims

To determine the annual incidence of DKA at initial presentation with type 1 diabetes mellitus in all children <15 years of age between 2000 and 2009 in the Auckland region.

## Methods

Data from Auckland children with newly diagnosed T1DM between 1 January 2000 and 31 December 2009 were collected from Starbase, the Starship Children’s Hospital diabetes database. T1DM was confirmed by the presence of glutamic acid decarboxylase and/or tyrosime phosphatise-like protein (IA2) antibodies. DKA was defined by international criteria as venous or capillary pH and bicarbonate as mild DKA with pH <7.30 and bicarbonate <15 mmol/l, moderate DKA pH <7.20 and bicarbonate <10 mmol/l and severe DKA ph <7.10and bicarbonate <5 mmol/l.

## Results

There were 481 children diagnosed with T1DM in the Auckland region (population 1,500,000) between 2000 and 2009. Over the study period the DKA incidence was highly variable (32 to 63% without a discernable change in incidence over the 10 year period [p=0.11]), thus data are expressed as a means over the study period 2000-2009. There were 47.4% of children in DKA at initial presentation which is very similar to the DKA incidence we reported for 1995-96 of 42%[1]. Of those with DKA in the current study 46.7% had mild, 22.0% moderate and 33.3% severe DKA. Younger age was associated with an increasing risk of DKA at 8% per year of age. Children <5 yrs of age had a much higher incidence of DKA at 62% compared to 43% in children 5-15 years of age. Neither sex, BMI, ethnicity nor socioeconomic status (assessed by NZDep score) influenced the likelihood of DKA.

## Conclusions

The incidence of DKA at initial presentation of type 1 diabetes mellitus is much higher in Auckland children compared to other published studies. DKA incidence has plateaued over the past 14 years and occurs far more frequently in young children. We speculate that improved community awareness of the symptoms of T1DM will lead to earlier diagnosis of T1DM and avoidance of DKA and associated sequelae.
